# Complexity and Competition in Appetitive and Aversive Neural Circuits

**DOI:** 10.3389/fnins.2012.00170

**Published:** 2012-11-26

**Authors:** Crista L. Barberini, Sara E. Morrison, Alex Saez, Brian Lau, C. Daniel Salzman

**Affiliations:** ^1^Department of Neuroscience, Columbia UniversityNew York, NY, USA; ^2^Department of Psychiatry and Behavioral Science, Albert Einstein College of MedicineBronx, NY, USA; ^3^Centre de Recherche de l’Institut du Cerveau et de la Moelle ÉpinièreParis, France; ^4^Department of Psychiatry, Columbia UniversityNew York, NY, USA; ^5^Kavli Institute for Brain Sciences, Columbia UniversityNew York, NY, USA; ^6^W. M. Keck Center on Brain Plasticity and Cognition, Columbia UniversityNew York, NY, USA; ^7^New York State Psychiatric InstituteNew York, NY, USA

**Keywords:** amygdala, orbitofrontal cortex, value processing, reward, punishment

## Abstract

Decision-making often involves using sensory cues to predict possible rewarding or punishing reinforcement outcomes before selecting a course of action. Recent work has revealed complexity in how the brain learns to predict rewards and punishments. Analysis of neural signaling during and after learning in the amygdala and orbitofrontal cortex, two brain areas that process appetitive and aversive stimuli, reveals a dynamic relationship between appetitive and aversive circuits. Specifically, the relationship between signaling in appetitive and aversive circuits in these areas shifts as a function of learning. Furthermore, although appetitive and aversive circuits may often drive opposite behaviors – approaching or avoiding reinforcement depending upon its valence – these circuits can also drive similar behaviors, such as enhanced arousal or attention; these processes also may influence choice behavior. These data highlight the formidable challenges ahead in dissecting how appetitive and aversive neural circuits interact to produce a complex and nuanced range of behaviors.

## The Importance of Learning to Predict Reinforcement for Punishment-Based Decision-Making

The decision-making process – arguably one of the most important “executive” functions of the brain – can be influenced by a variety of different types of information and motivators. Punishment-based decisions constitute an important subcategory that is common to a wide phylogenetic range, from nematodes to rodents to humans. Studies old and new have shown that punishment engages brain systems specialized for processing aversive information (Seymour et al., 2007). Historically, these systems have been studied most frequently in rodents, and this work has revealed many aspects of the neural mechanisms driving behavior elicited by the threat of aversive stimuli (Davis, 1992; LeDoux, 2000). In everyday life, however, decisions typically require integrating information about potential punishments *and* rewards, as well as myriad factors such as external environment and internal drives. This is especially true in primates, as they exhibit particularly complex behavioral repertoires.

Rewards and punishments are reinforcers with opposite valence (positive versus negative), and they often drive behavior in opposite directions – e.g., approaching a rewarding stimulus or avoiding a threat. Moreover, punishment-based decisions are often made in a context in which rewards and punishments are both possible consequences of an action; therefore, brain systems processing aversive information must interact with brain systems processing rewards – interactions that presumably underlie how punishments and rewards compete to drive behavior and decision-making. Scientists have long appreciated these facts and have often posited that appetitive and aversive systems operate in an “opponent” manner (Konorski, 1967; Solomon and Corbit, 1974; Dickinson and Dearing, 1979; Grossberg, 1984; Daw et al., 2002). However, appetitive and aversive stimuli also have certain common attributes – e.g., they are both usually more salient than non-reinforcing stimuli – and thus appetitive and aversive systems need not always act in opposition to each other. Rather, stimuli of both valences may mediate a number of processes, such as enhanced arousal or enhanced attention to stimuli predictive of reinforcement (Armony and Dolan, 2002; Anderson, 2005; Lang and Davis, 2006; Phelps et al., 2006; Brosch et al., 2008; Ilango et al., 2010; Pinkham et al., 2010; Anderson et al., 2011).

Punishment-based decisions are generally choices that are based on one or more prior experiences with an aversive outcome. Typically, an organism learns that a sensory cue predicts a possible negative outcome – e.g., the taste of spoiled food precedes illness – and later must decide what to do to avoid or defend against that outcome. Thus, learning to anticipate negative outcomes is an essential skill for subsequently being able to make optimal decisions in the face of possible punishment. This is also true for rewards: the adaptive response is to acquire the reward, rather than avoid it, but anticipation is critical in both cases.

Because accurately predicting reinforcement – whether punishment or reward – plays such a vital role in decision-making, our work has focused on understanding the neurophysiological processes whereby the brain comes to predict reinforcement as a result of learning. We have sought to understand where and how signals in the brain represent anticipated positive or negative outcomes, and whether those signals occur at a time and in a manner such that they could be used as input to decision-making processes. We have often referred to these signals as *value* signals. Although our published studies have not characterized these signals during an explicit decision-making task, the tasks we employed do provide measures that appear to co-vary with the amount and type of the reinforcement associated with a stimulus (Paton et al., 2006; Belova et al., 2007, 2008; Salzman et al., 2007; Morrison and Salzman, 2009, 2011; Morrison et al., 2011). We believe that the *value* of anticipated possible outcomes often drives behavior, and the estimation of value may be computed on-line during decision-making by taking into account expected potential reinforcement as well as a variety of internal variables (e.g., hunger or thirst) and external variables (e.g., how difficult a reward would be to acquire; Padoa-Schioppa, 2011). We refer to the circuits that process and generate appetitive and aversive reinforcement predictions as value processing circuits, although in some cases work remains to be done to understand how different internal and external variables impact representations of reinforcement predictions.

Where in the brain does processing about reinforcement predictions occur? Early work indicated that the amygdala, a key structure in the limbic system, plays a central role in processing one of the primary negative emotions, the fear elicited by a stimulus predicting aversive consequences. Seminal fear conditioning studies in rats found that both learning and memory of fearful events required an intact, functional amygdala (Davis, 1992; LeDoux, 2000; Maren and Quirk, 2004). Since then, it has become clear that the purview of the amygdala extends beyond fear to include other emotions, including positive ones (Holland and Gallagher, 1999; Baxter and Murray, 2002; Everitt et al., 2003; Paton et al., 2006; Belova et al., 2008; Morrison and Salzman, 2010; Salzman and Fusi, 2010). These results suggest that the amygdala may carry signals related to the computation of both positive and negative value.

How do amygdala signals come to impact behavior? The amygdala is heavily interconnected with many other areas of the brain, providing an array of anatomical pathways by which it can participate in learning and decision-making. It receives input from multiple sensory modalities (McDonald, 1998; Amaral et al., 2003; Freese and Amaral, 2005), which accords with the amygdala’s established role in associative learning; information from predictive sensory cues converges with input about reinforcing outcomes at the single cell level (e.g., Romanski et al., 1993). Furthermore, lesions of the amygdala impair reinforcer devaluation (Baxter and Murray, 2002; Izquierdo and Murray, 2007), indicating that the amygdala plays a role not only in learning reinforcement contingencies, but also in adjusting these representations as the value of associated reinforcement outcomes changes.

Although the amygdala participates in learning stimulus-reinforcement associations that in turn may be utilized and adjusted during decision-making, it does not act alone in these processes. The amygdala has reciprocal connections with orbitofrontal cortex (OFC; McDonald, 1991; Carmichael and Price, 1995; Stefanacci and Amaral, 2000, 2002; Ghashghaei et al., 2007), a cortical area thought to play a central role in value-based decisions (Padoa-Schioppa and Assad, 2006; Wallis, 2007; Padoa-Schioppa, 2011). OFC may be important for implementing executive or cognitive control over behavior, and endowing subjects with the ability to rationally analyze their options, as well as to tune their behavior to what is socially acceptable in the face of emotionally driven impulses (Damasio, 1994; Rolls, 1996; Bechara et al., 2000; Berlin et al., 2005; Ochsner and Gross, 2005). Part of this may be due to the fact that OFC seems to play a role in the simple ability to anticipate aversive stimuli or negative outcomes, as well as positive outcomes (Tremblay and Schultz, 2000; Roberts et al., 2004; Young et al., 2010).

In this paper, we review our efforts to understand the roles of the amygdala and OFC in acquiring representations of reinforcement contingencies. As we reviewed above, these representations may be critical substrates for reward-based and punishment-based decision-making. One of the striking findings in these investigations concerns the differential dynamics of processing that takes place in appetitive and aversive systems in amygdala and OFC. The amygdala appears to have evolved an aversive system that learns changes in reinforcement contingencies more rapidly than its counterpart in OFC; but, for appetitive networks, the time courses of learning in the two brain areas are reversed. Moreover, both single unit and local field potential (LFP) data point to complex interactions between amygdala and OFC that change as a function of learning. Although appetitive and aversive systems have been posited to act in an opponent manner, this complex pattern of interactions suggests that a more nuanced framework may be required to understand the relative contribution of these networks during learning and decision-making. Moreover, behavioral evidence indicates that appetitive and aversive stimuli can have a variety of effects on cognitive processes, some of which may be induced by stimuli of either valence. Altogether, these data suggest that appetitive and aversive systems may act in congruent *and* opponent fashions – even at the same time – and do not merely compete to determine the most valuable behavioral option during decision-making.

### Positive and negative cells in the brain

We have focused on trying to understand neural circuits involved in punishment and aversive learning, and how these circuits may differ from and interact with circuits involved in rewards and appetitive learning. When we began our experiments several years ago, only a few studies had examined the neurophysiology of the amygdala in primates (Sanghera et al., 1979; Nishijo et al., 1988, 2008; Rolls, 2000; Sugase-Miyamoto and Richmond, 2005; Wilson and Rolls, 2005). Furthermore, no primate lab had undertaken simultaneous recordings in amygdala and OFC to understand dynamic interactions between the brain structures during learning.

Our experimental approach strove to disambiguate neural responses that might be related to the sensory properties of visual conditioned stimuli (CSs) from responses related to the reinforcement contingencies. To accomplish this, we used a mixed appetitive/aversive reversal learning paradigm. This paradigm combined a conditioning procedure with standard extracellular physiology in rhesus monkeys; we measured the physiological responses of individual neurons to CSs that signaled an impending positive or negative US. CSs were small fractal patterns, positive outcomes were small aliquots of water, and negative outcomes were brief airpuffs directed at the face (Paton et al., 2006; Belova et al., 2007, 2008; Morrison and Salzman, 2009, 2011; Morrison et al., 2011). In these experiments, one CS was initially paired with reward and another with an aversive stimulus (unconditioned stimuli, USs); then, without warning, we reversed the reinforcement contingences of the CSs. We recorded single neuron responses while monkeys learned the initial CS-US associations and their reversal. One major advantage of this approach was that reinforcements – particularly aversive “punishments” – were unavoidable, so we were able to unequivocally identify neural activity related to the anticipation of appetitive and aversive reinforcement.

**Figure 1:**
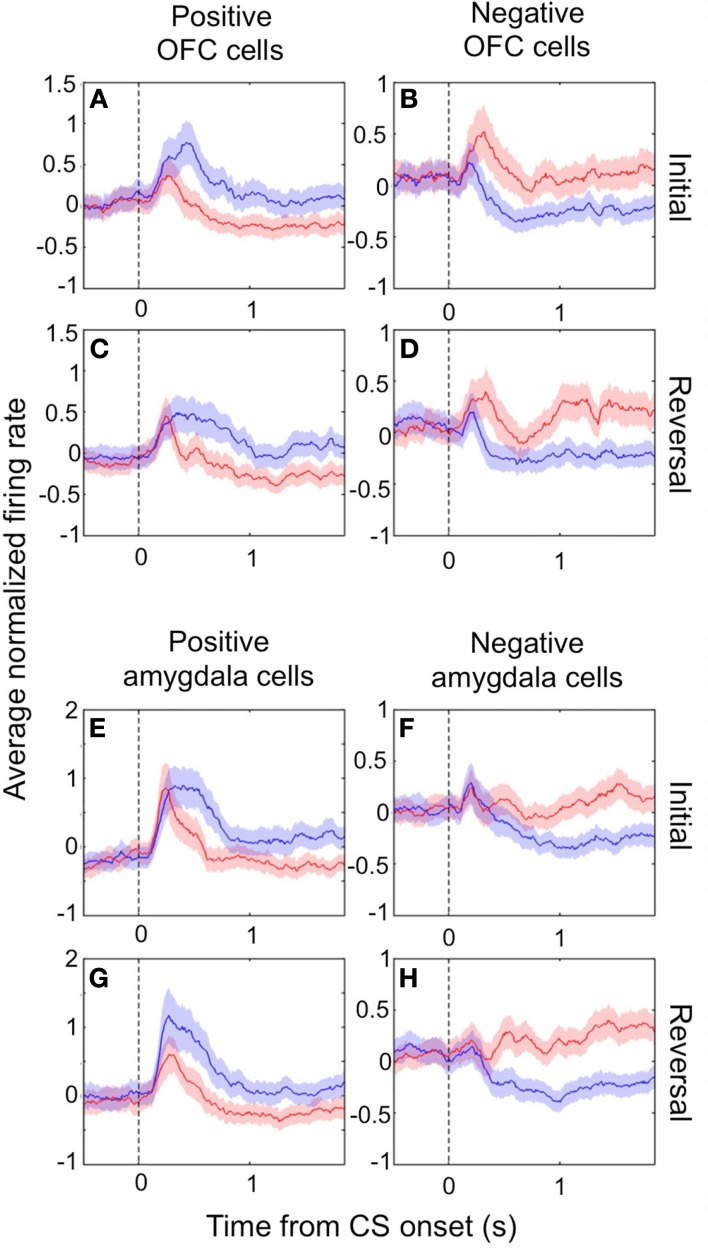
**Value-coding cells in the amygdala and OFC**. The average normalized neural activity (±SEM) as a function of time since CS onset is shown for the population of positive value-coding cells **(A,C,E,G)** and negative value-coding cells **(B,D,F,H)**, in OFC **(A–D)** and the amygdala **(E–H)**. Responses are shown before **(A,B,E,F)** and after **(C,D,G,H)** reversal of the outcome contingencies associated with each CS. Peristimulus time histograms (PSTHs) were built using 10 ms non-overlapping bins, *Z*-scoring, and then averaging cells together, and lastly smoothing by calculating a 10-bin moving average. Blue lines, positive CS trials; red lines, negative CS trials. Vertical dotted line, CS onset. Adapted from Morrison et al. (2011), Figure 3, with permission.

In both the amygdala and OFC, we observed two populations of neurons that fired more for positive or negative outcomes, respectively, which we refer to as positive and negative value-coding cells. The response profiles for these two populations are shown in Figures 1A–D for OFC and in Figures 1E–H for the amygdala. Shortly after CS onset, both cell populations systematically fire differentially for CSs paired with positive or negative reinforcement. Reversing the reinforcement contingencies (Figures 1C,D,G,H for positive and negative cells, respectively) demonstrates that the differential firing is specifically related to the reinforcement contingencies and not other aspects of the CS, such as specific visual features. Note that after reversal, an image formerly associated with a reward now leads to a punishment, and vice-versa; after only a few trials of exposure to these new contingencies (Paton et al., 2006; Belova et al., 2007; Morrison et al., 2011), the neural response pattern shifts to reflect these changes, such that the response profiles look quite similar before and after reversal.

The encoding of reinforcement contingencies seems to reflect the overall motivational significance, or *value*, of a US associated with a CS, and not other types of information learned during conditioning. Several lines of evidence support this conclusion. First, neither amygdala nor OFC neurons encode motor responses elicited by USs on our task, indicating that neurons do not appear to represent the relationship between a CS and the motor response elicited by USs (Paton et al., 2006; Morrison and Salzman, 2009). Second, both OFC and amygdala neurons generally do not simply represent the relationship between a CS and the sensory qualities of a preferred US. Rather, we found that OFC and amygdala neurons respond in a graded manner to CSs predicting large rewards (LRs), small rewards (SRs), and negative outcomes; this means that a cell that prefers a CS associated with an aversive airpuff also responds differentially to CSs associated with water rewards, and thus encodes information about two types of outcomes. Moreover, since the outcomes include two modalities (taste and touch), it is unlikely that the neural response is primarily driven by a physical quality of one type of outcome, such as the strength or duration of the airpuff (Belova et al., 2008; Morrison and Salzman, 2009).

Third, positive and negative neurons often appear to track value in a consistent manner across the different sensory events in a trial – including the fixation point, CS, and US presentations – even though those stimuli differ in sensory modality. This has led us to suggest that amygdala and OFC neurons represent the overall value of the animals’ “state,” or situation (Belova et al., 2008; Morrison and Salzman, 2009, 2011). Finally, in an additional series of experiments that examined the representation of “relative” value in different contexts, amygdala neurons changed their firing rate in accordance with changes in the relative value of a CS, even when the absolute value (i.e., reward size) of the associated US does not change (Schoer et al., 2011). This phenomenon has also been observed in the OFC (Padoa-Schioppa, 2009; Schoer et al., 2009).

In contrast to the signals just described, there are doubtless other signals in the brain that encode the magnitude of single stimulus dimensions – e.g., the size or taste of specific rewards. However, these signals would not, in and of themselves, be sufficient to inform choices made between outcomes that were in different modalities.

### Dynamics during learning

The neurons we describe provide a dynamic representation that changes rapidly during learning. Overall, during reversal learning, the change in the neural responses in both amygdala and OFC was on a timescale similar to changes in the monkey’s behavior. Behavioral metrics of the monkey’s expectation – anticipatory licking of the water tube preceding rewards and anticipatory “blinking” before aversive airpuffs – reversed within a few trials, indicating that monkeys learned the new associations quite rapidly (Paton et al., 2006; Morrison et al., 2011). Amygdala and OFC neural activity likewise began to change their responses to CSs within a few trials of a reversal in reinforcement contingencies (Paton et al., 2006; Belova et al., 2007; Morrison et al., 2011). This sequence of neural and behavioral changes indicates that the amygdala and OFC could be involved in the monkeys’ learning of new reinforcement contingencies.

**Figure 2:**
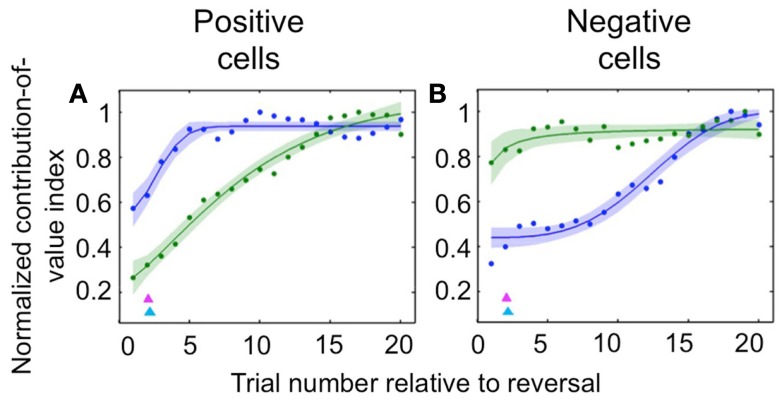
**Comparison of the time courses of learning-related activity in positive and negative value-coding neurons in the amygdala and OFC**. Normalized average contribution of image value to neural activity, derived from ANOVA, plotted as a function of trial number after reversal for positive value-coding neurons **(A)** and negative value-coding neurons **(B)**. Blue lines, OFC; green lines, amygdala; red and cyan arrowheads, mean licking and blinking change points, respectively. Adapted from Morrison et al. (2011), Figures 5C,D, with permission.

Neuroscientists have long believed that the prefrontal cortex, and OFC in particular, drives reversal learning (Iversen and Mishkin, 1970; Thorpe et al., 1983; O’Doherty et al., 2001; Schoenbaum et al., 2002; Chudasama and Robbins, 2003; Fellows and Farah, 2003; Hornak et al., 2004; Izquierdo et al., 2004; Chamberlain et al., 2008; Hampshire et al., 2008; Ghahremani et al., 2010); but some have recently proposed that in fact representations in OFC may update more slowly upon reversal than those elsewhere (Schoenbaum et al., 1998, 2003; Saddoris et al., 2005). Because we recorded amygdala and OFC activity simultaneously, we were able to examine the dynamics of learning in positive and negative value-coding neurons in both amygdala and OFC in order to characterize their relative timing. We found that appetitive and aversive networks in OFC and amygdala exhibited different learning rates, and – surprisingly – that the direction of the difference depended on the valence preference of the cell populations in question. For positive cells, changes in OFC neural activity after reversal were largely complete many trials earlier than in the amygdala; for negative cells, the opposite was true (Figure 2). In each case, the faster-changing area was completing its transition around the time of the onset of changes in behavior; meanwhile the other, more slowly changing area did not complete the shift in firing pattern until many trials after the behavioral responses began to change. Thus, signals appropriate for driving behavioral learning are present in both brain structures, with the putative aversive system in the amygdala and appetitive system in OFC being particularly sensitive to changes in reinforcement contingencies. This finding may reflect the preservation across evolution of an aversive system in the amygdala that learns very quickly in order to avoid threats to life and limb.

### During versus after learning

**Figure 3:**
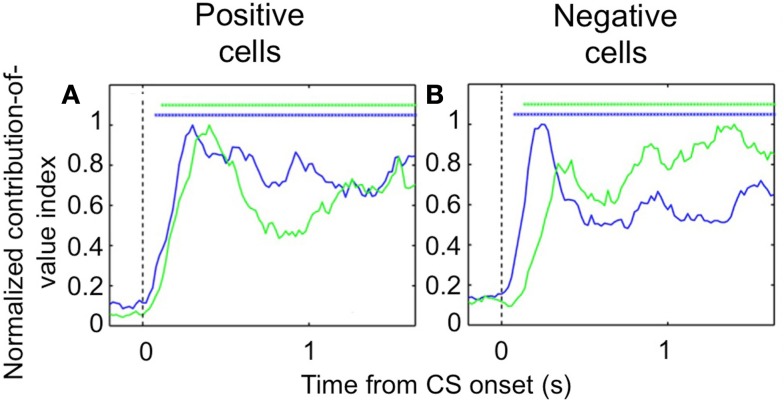
**Encoding of image value in OFC and the amygdala**. The contribution of image value as a function of time for positive value-coding cells **(A)** and negative value-coding cells **(B)**. Asterisks, time points at which the average contribution of value is significant (Fisher *p* < 0.0001) for OFC (blue lines) and the amygdala (green lines). Vertical dotted line, CS onset. Adapted from Morrison et al. (2011), Figures 8E,F, with permission.

Despite the complex pattern of dynamics we observed during learning, once the new CS-US contingencies have been established, we found that *both* populations of OFC cells – positive value-coding and negative value-coding – predict reinforcement earlier in the trial than their counterparts in the amygdala (Figure 3). To demonstrate this, we examined trials after learning had taken place and determined the earliest point in the trial each area begins to significantly differentiate between images that predict reward and images that predict airpuff. For both positive and negative cell populations, OFC predicted reinforcement more rapidly after image onset. Thus, it appears that the relationship between single unit firing in the appetitive and aversive networks in the two brain areas evolves as a function of learning, with the OFC perhaps assuming a more primary role after learning.

**Figure 4:**
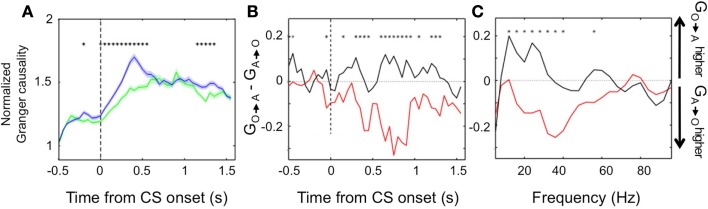
**Granger causality between the amygdala and OFC**. **(A)** Average normalized Granger causality (±SEM) for the OFC-to-amygdala direction (blue) and the amygdala-to-OFC direction (green). For each pair of OFC-amygdala LFP recordings, Granger causality was computed for all trials after reversal, then averaged across pairs. Only pairs with significant Granger causality at some point during the trial were included in the average, which combines frequencies from 5 to 100 Hz. Asterisks, bins with significantly different causality for the two directions (permutation test, *p* < 0.05). **(B,C)** Granger causality changes with learning. The difference between the mean Granger causality in the two directions (subtracting amygdala-to-OFC from OFC-to-amygdala) was separately calculated for early (during learning, red line) and late (post-learning, black line) trials after reversal. This comparison is shown for all frequencies 5–100 Hz as a function of time within the trial **(B)** and for the CS and trace intervals combined as a function of frequency **(C)**. Asterisks, bins where the difference between during-learning and post-learning values was significant (permutation test, *p* < 0.05). Adapted from Morrison et al. (2011), Figure 9, with permission.

We found further evidence of the evolving dynamic relationship between amygdala and OFC during learning by examining LFP data recorded during the reversal learning task. To do so, we applied Granger causality analysis, which measures the degree to which the past values of one neural signal predict the current values of another (Granger, 1969; Brovelli et al., 2004), to the simultaneously recorded LFPs in the amygdala and OFC. Remarkably, we found significant Granger causality in *both* directions that increased upon CS onset (Wilcoxon, *p* < 0.01; Figure 4A). Notably, during learning, Granger causality was stronger in the amygdala-to-OFC direction, but after learning, Granger causality was strongest in the OFC-to-amygdala direction (Figures 4B,C). This result is consistent with single unit data showing that, after reversal learning has occurred, OFC predicts reinforcement with a shorter latency after CS onset. This positions the OFC to be able to drive or modulate amygdala responses to value-laden CSs after learning. (Note, however, that the amygdala continues to be able to influence processing in OFC, just not as strongly as the reverse.).

### Conflict within appetitive and aversive circuits

There is an additional level of complexity within appetitive and aversive circuits that has not received much attention on the physiological level, namely competition and conflict within these circuits. Our learning data suggest that the signals carried by different neural circuits may be updated at different rates in different brain areas. This suggests that these systems might at times conflict with each other. Another possible example of competition is that between executive areas – which allow us to evaluate potential outcomes on a practical and rational level – and limbic areas, which are more involved in emotional processing, and which might provide a value signal based more heavily on immediate sensory experience and emotion-laden associations. For example, the amygdala and OFC themselves may at times “recommend” different responses, the former mediating more emotionally driven responses and the latter more executive or cognitive behaviors (De Martino et al., 2006).

This phenomenon has been given some attention on the behavioral level (McNeil et al., 1982; Damasio et al., 1994; Kahneman and Tversky, 2000; Loewenstein et al., 2001; Greene and Haidt, 2002), and has also been examined using fMRI in humans (McClure et al., 2004, 2007; De Martino et al., 2006; Kable and Glimcher, 2007). However, few studies have examined appetitive and aversive circuits at the level of single cells during a decision-making task involving rewards and punishments. To best investigate the interactions between appetitive and aversive neural circuits, such a decision-making task should include conditions in which rewards and aversive stimuli must be weighed against each other in order to guide behavior. As a first step, we trained monkeys to perform a simple two-choice task involving rewards and aversive stimuli (described below). We discovered that, even on this simple task, behavioral choices appear to be influenced not only by the value of the reinforcement associated with cues, but also by the salience of cues.

**Figure 5:**
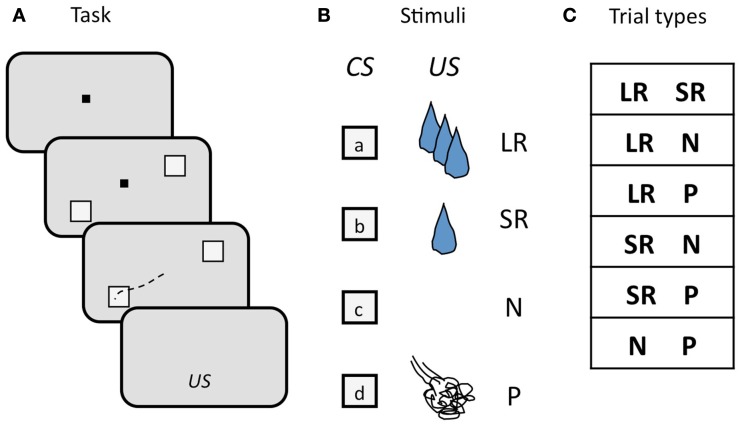
**Schematic illustration of the design of the two-choice task**. **(A)** Sequence of events in each trial. The monkey begins each trial by foveating a central fixation point (FP, black square), then two visual targets appear, straddling the FP, a delay ensues, the FP goes out, and the monkey makes an eye movement (black dashed line) to one of the two targets to select it. Targets are extinguished, and, after another short delay, the associated outcome (US) is delivered. **(B)** Visual targets (CSs) and associated outcomes (USs). Four targets are used as CSs, each one associated with one of the four possible USs. CSs are random grayscale stick figures (not shown); USs: LR, large reward; SR, small reward; N, neutral; P, punishment. **(C)** Trial types, determined by the outcome of the two CSs offered. CSs were counter-balanced for location.

We used a two-choice task in which monkeys selected visual targets by making a saccade to the target of their choice. Monkeys viewed two visual targets on each trial, each of which was a CS associated with a particular outcome. After maintaining fixation during a 900–1200 ms delay period, monkeys chose one of the two targets by foveating it, followed by delivery of the associated outcome (Figure 5A). There were four possible outcomes: a LR, a SR, no reinforcement (N), or a punishment (P), where rewards were small amounts of water and punishments were brief airpuffs directed at the face. The four CSs (one for each outcome; Figure 5B) were offered in all possible combinations, with the exception of two of the same kind. Trial conditions were pseudo-randomly interleaved, and counter-balanced for spatial configuration. The list of trial types is shown in Figure 5C. New sets of CSs were used in each session. Two independent stimulus sets were used, and trials drawing from the two sets were interleaved in pseudo-random order. In each session, a pair of locations on the monitor was chosen and used for the duration of the session. The locations varied, but each pair always straddled the fixation point. While monkeys were free to choose either target, they had to make a choice: incomplete trials were repeated until one or the other target was chosen.

**Figure 6:**
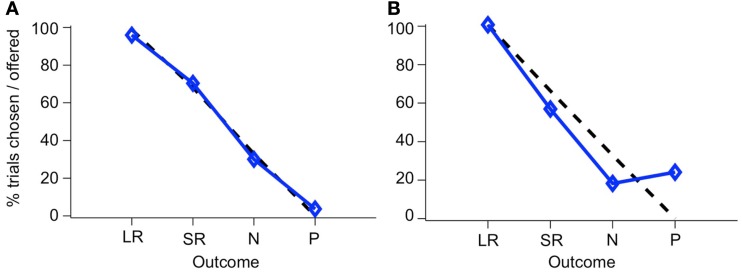
**Choice behavior in the two-choice task**. The percent of trials that a CS was chosen when it was offered is shown for each CS. Blue line, monkey’s choices; dashed black line, optimal behavior. Choice behavior is shown for two sessions, one where the monkey rarely chose the P target **(A)**, and one where he chose it frequently **(B)**. The two stimulus sets have been combined in this figure.

If monkeys always chose the higher-value target, then plotting the percent of trials on which a CS was chosen, out of all trials on which that CS was offered, yields a straight line, since LR is always the higher-value target when presented, SR on 2/3 of trials when presented, N on 1/3 of trials, and P on no trials, as can be seen in the list of trial conditions (see Figure 5C). We will refer to this as “optimal” behavior. In Figure 6, two example sessions are shown. The first is a session in which a monkey chose the higher-value target most of the time, such that the plot of the number of times each target was chosen follows the optimal behavior line quite closely (Figure 6A). In the second example, however, the same monkey chose the punished target many times, and about as often as he chose the neutral (non-reinforced) target (Figure 6B).

**Figure 7:**
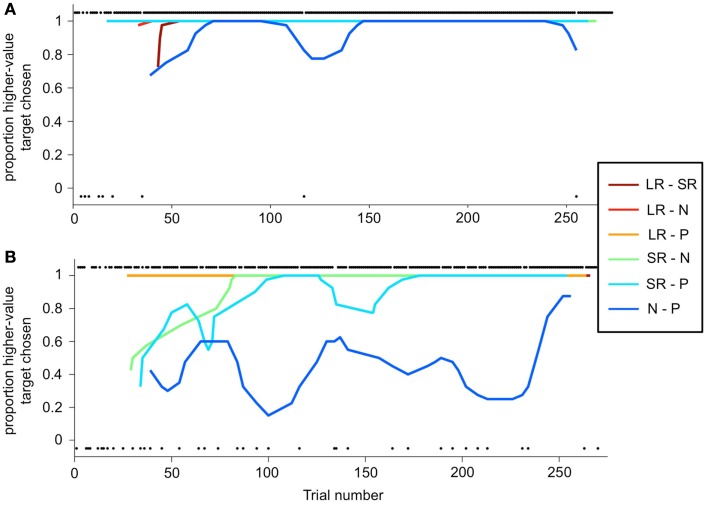
**Choice behavior as a function of trial number**. **(A,B)** A running average is calculated (6-trial boxcar) for each trial type (the two stimulus sets are again folded together), as a function of trial number within the session, for the same sessions shown in Figure 6. Choice behavior on each trial is calculated as the proportion of higher-value targets chosen, and on each trial is either 1 (higher-value target was chosen) or 0 (lower-value target was chosen). Individual black dots show when one outcome or the other was chosen on a per-trial basis. Thus, dots along the bottom of the figure indicate a lower-value choices. Dots are offset from 0 to 1 for clarity. Running average lines start at different trial numbers because they start on the *n*th trial for that trial type, where *n* is the width of the running average, but are plotted against actual trial number in the session.

The deviation from optimal behavior seen in Figure 6B is not due to an overall drop in performance, but to a change in behavior on a single trial type: the N-P stimulus pair. In Figure 7, a running local average of the proportion of trials on which the monkey chose the higher-value target is shown, broken down by trial type, for the same two sessions shown in Figure 6. When offered a choice between a reward and a punishment, the monkey reliably chose the reward (LR-P and SR-P trial types in Figures 7A,B). However, when offered a choice between no reinforcement and a punishment, in some sessions, the monkey chose punishment quite often (N-P trial type in Figure 7B). These two sessions are representative of the type of choice behavior we observed.

This choice pattern was perplexing to us at first. We noticed that sometimes monkeys avoided the punished target in a session, while other times he chose it over the neutral target a substantial fraction of the time. We checked and manipulated a number of parameters: did monkeys find the punishment aversive? Was it aversive *enough*? Did monkeys understand the CS-US contingencies? What we found, in two monkeys, was an abundance of evidence that subjects *did* understand the task contingencies, *did* find the airpuff aversive, and yet chose the punished target despite the aversive outcome they knew would follow. Evidence in support of the idea that the airpuff was indeed aversive included: visible frustration and displeasure upon airpuff delivery, defensive blinking behavior in anticipation of airpuff, statistically significant greater likelihood of breaking fixation on N-P trials, and willingness to work being clearly dependent on the strength or frequency of airpuff delivery, with increases in any of these variables quickly leading to the monkey’s refusing to work for the rest of the day. None of these were observed in relation to rewarding outcomes.

**Figure 8:**
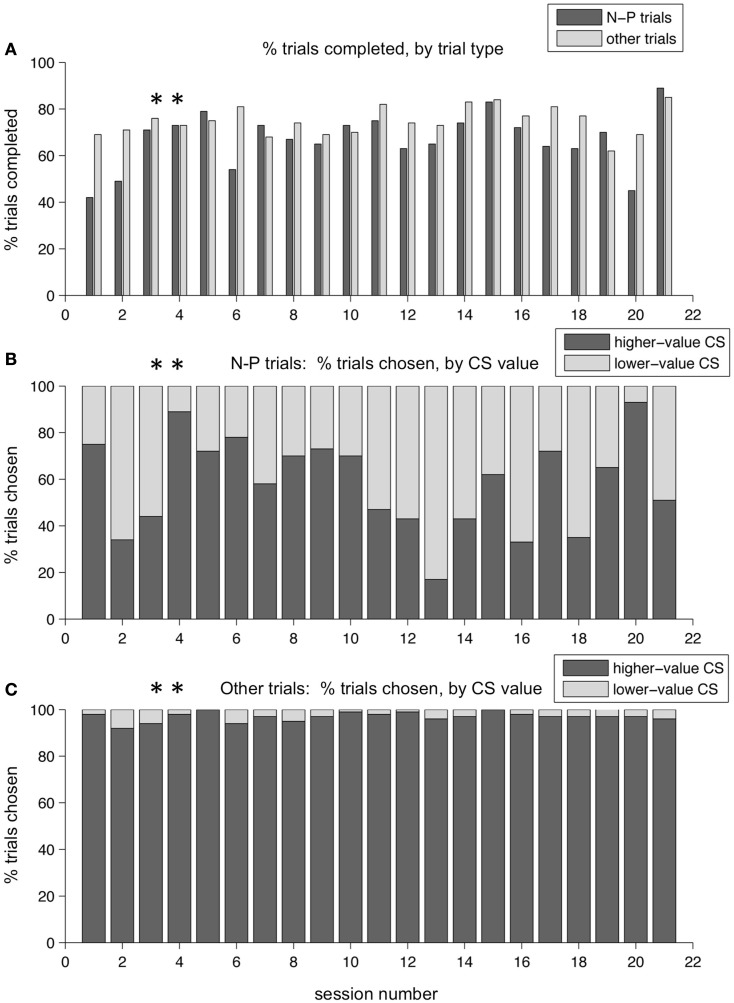
**Choice behavior in the two-choice task over time**. Performance over a training period of weeks for one monkey. **(A)** The percent of trials completed is shown, for each session, for N-P trials and all other trials separately (dark and light gray bars, respectively). **(B)** The percent of N-P trials, for each session, on which the monkey chose N (higher-value CS, dark gray bars) or P (lower-value CS, light gray bars). **(C)** The percent of other trial types, for each session, on which the monkey chose the higher-value target (dark gray bars) or the lower-value target (light gray bars). Asterisks mark the two sessions shown in Figures 6 and 7.

Over a period of training lasting several months, these patterns persisted. Figure 8 shows the performance across a series of sessions over a period of a few weeks in one monkey. The two example sessions shown in the previous figures are marked with asterisks. In Figure 8A, the percent of trials completed for N-P versus other trial types is displayed. On average, the monkey broke fixation before completing the trial more often on N-P trials than on other trial types – resulting in a lower percent of trials completed – which is indicative of that trial type being aversive, difficult, or both. (Note that the two sessions shown in Figures 6 and 7 are not representative of this overall pattern, having lower than average percent break-fixation trials). Figure 8B shows the percent of trials on which the monkey chose the N-target on N-P trials (dark gray bars, %N of NP) as compared to choosing the P target (light gray bars). What is apparent is that %N of NP varied day to day, and did not appear to plateau at a stable level, nor was there a trend in either direction as training progressed. Note that the selection of the punished target on N-P trials occurred during blocks in which, on all other interleaved trial types, the monkey chose the higher-value target nearly all of the time (Figure 8C). This same pattern was seen in other training periods for this monkey, as well as across all training periods in the second monkey.

**Figure 9:**
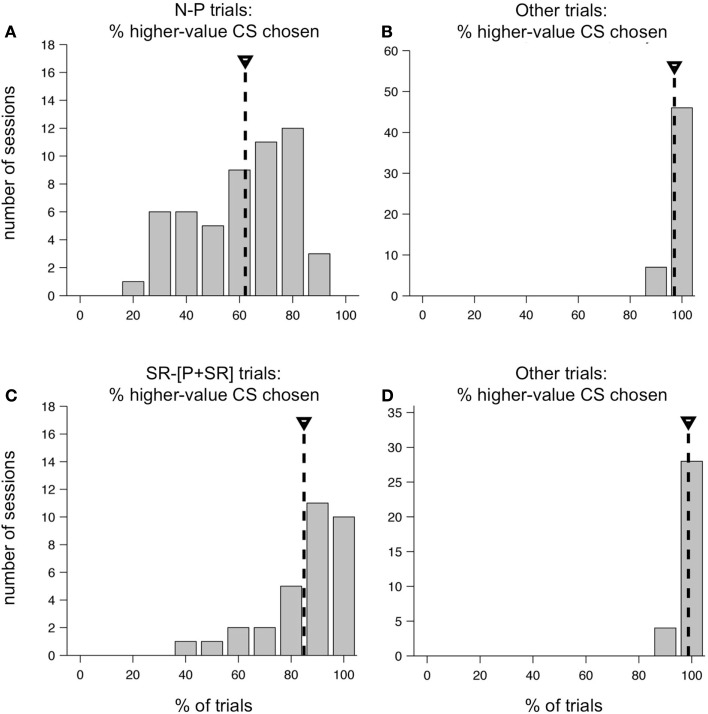
**Distribution of higher-value target choices in two versions of the two-choice task**. Performance of one monkey in the original two-choice task **(A,B)** and the modified two-choice task **(C,D)**. **(A)** Distribution of the percent of N-target choices on N-P trials across all sessions in a 5 month training period. Mean, 62.2% (mean > 50%, *t*-test, *p* < 0.0001). **(B)** Distribution of the percent higher-value choices on non-N-P trial types across the same set of sessions as in **(A)**. Mean, 97.1% (mean > 50%, *t*-test, *p* < 0.0001). **(C)** Distribution of the percent of SR-target choices on SR−[P + SR] trials across 32 sessions. Mean, 84.9%, (mean > 50%, *t*-test, *p* < 0.0001). **(D)** Distribution of the percent higher-value choices on non-SR−[P + SR] trial types across the same set of sessions as in **(C)**. Mean, 98.7%, (mean > 50%, *t*-test, *p* < 0.0001).

On average, one monkey chose neutral CSs over punished CSs only slightly more than half the time. Figure 9A shows the distribution of %N of NP across all training sessions, including the subset shown in Figure 8. The mean was 62.2%, and was significantly greater than 50% (*t*-test, *p* < 0.0001). This was over a training period of 5 months, and after trying a host of manipulations to ensure that the monkey understood the task and the CS-US contingencies involved. Also, note that on interleaved trials, the monkey was choosing the higher-value target virtually all the time (Figure 9B). In the second monkey, the average %N of NP was very close to 50%, and was not statistically significant (mean, 50.4%, mean > 50%, *t*-test, *p* = 0.4409), even though that monkey was also trained extensively and exposed to the same set of task manipulations as the first monkey. However, his performance on other trial types was similarly very high (mean, 89.1% higher-value target chosen, mean > 50%, *t*-test, *p* < 0.0001).

While there are several possible explanations of this counter-intuitive behavior, we favor one explanation that fits with some of the other examples of neural systems in competition. In particular, we believe that on the N-P trial type, the salience and value of cues were in conflict, and this conflict pushed monkeys toward different choices. This was not true on any of the other trial types, in which the most salient CS on the screen was also the most valuable (whatever the highest level of reward was). On N-P trials, however, the N-target is more valuable than the P target (presumed zero value versus negative value), but the P target, by virtue of its association with an aversive airpuff, is very likely to be more salient. Thus the P target is chosen some of the time, even though it is not necessarily what monkeys prefer, due to a strong impulse to foveate – i.e., look at or orient toward – this highly salient, behaviorally relevant stimulus. Further evidence to support this idea is that monkeys were much more indecisive on N-P trials than on other trials: this was apparent in the percentage of break-fixation trials (Figure 8A), and in the observation that monkeys often looked quickly back and forth between targets, even though this behavior led to a greater number of incomplete trials. The monkeys did not do this on other trial types. As might be expected for trial types that are more difficult or less certain, monkeys exhibited greater spatial bias on N-P trials than on other trial types. The differences were modest: first monkey, 10.0% versus 1.6% bias, and second monkey, 8.3% versus 2.4% bias, for N-P and other trials, respectively, when measured across all sessions. (Bias is the percentage over 50% that a preferred spatial location is chosen; a 10% bias is equivalent to a location being chosen 60% of the time). While both differences were statistically significant (*t*-test, *p* < 0.0001 in both cases), the small magnitude indicates that other factors had a strong impact on the monkeys’ choices.

We suspected that the absence of a possible reward on N-P trials was having a major impact on the choice behavior of our monkeys. Therefore, we redesigned the task for the first monkey so that all outcomes included some level of reward, using as our set of possible outcomes: LR, SR, and a compound outcome of airpuff and SR (P + SR). For the compound outcome, the punishment was delivered first, followed by a short delay and then the SR. This change resulted in a substantial shift in the monkey’s behavior. Within a few training sessions, the monkey learned the new task and began consistently choosing the higher-value target most of the time on all trial types. At the beginning of each session, new CSs were introduced, and the monkey learned them within a small number of repetitions, and then chose the higher-value target virtually all of the time for the rest of the session. The monkey performed at this level consistently day after day: the average choice %SR on the trial type SR−[P + SR] was 84.9% (Figure 9C), which was significantly greater than 50% (*t*-test, *p* < 0.0001), and variations around this mean were much smaller than they had been in the first version of the task. As before, on all other trial types, which were interleaved, the monkey chose the higher-value target virtually all of the time (Figure 9D).

We have here, then, an example of counter-intuitive choice behavior that is robust and occurs when no reward is possible. As we mention above, we suspect that this is due to competition between the neural circuits processing value and salience; we would also speculate that the salience of negative outcomes only grows large enough to compete with value signals driving behavior when the value of the alternative outcome is small or zero (e.g., when a cue predicts no reinforcement). Clearly, this results in sub-optimal choice behavior. This is consistent with other studies that have noted sub-optimal performance in tasks where monkeys are forced to make a choice between outcomes and the greatest possible reward is very small or zero. For example, Peck et al. (2009) observed more incorrect choices on “neutral” as opposed to rewarded trials, and Amemori and Graybiel (2012) observed longer reaction times and more omission errors on a “reward–reward” control task when reward size was very low. Moreover, Amemori and Graybiel (2012) designed their main experimental task to include a SR for any choice because they found it necessary to “maintain motivation to perform the task.” The paradigm employed by Amemori and Graybiel differed from ours in a number of ways, including the use of a joystick movement operant response, limiting our ability to make a direct comparison of the behavior observed in the two tasks. On the other hand, our use of an eye movement operant response may have increased the efficacy by which representations of salience modulated behavior. There is good reason to believe that salience has privileged access to the oculomotor system (Bisley et al., 2004; Hasegawa et al., 2004), especially in the highly visually oriented primate, to promote rapid foveation of salient stimuli.

We suggest that our behavioral results may be an example of a competition between limbic and cortical circuits dedicated to emotional versus cognitive processing, respectively. This paradigm, in the macaque, may test the limits of the amount of cognitive control monkeys are able to exert over reflexive behaviors. While the monkey does succeed in overriding the impulse to look at the punished target some of the time, he does not do so all of the time. Humans, with their greater level of cognitive processing and control, would presumably have much less difficulty avoiding the punished target.

## Summary and Challenges

To make a decision, we often must predict how particular stimuli or courses of action lead to rewards or punishments. The ability to make these predictions relies on our ability to learn through experience the relationship between stimuli and actions and positive and negative reinforcement. It is therefore important to understand the representation of aversive and appetitive outcomes in the brain, both during and after learning, in order to understand how these signals generate behavior. However, at the same time, it’s important to recognize that the impact of appetitive and aversive circuits is not limited to behavior that is specific to the valence of the looming reinforcement. Activation of appetitive and aversive circuits can also elicit valence non-specific responses, such as enhanced arousal or attention.

A number of the studies in our lab have been directed at trying to understand the nature of appetitive and aversive circuits in the brain. Although there hadn’t been a great deal of work examining aversive processing at the physiological level in non-human primates in the past, some older studies suggested that our approach would be fruitful (e.g. Nishijo et al., 1988; Mirenowicz and Schultz, 1996; Rolls, 2000; Yamada et al., 2004). Our neurophysiological studies have expanded on these initial findings to create a more detailed picture of appetitive and aversive circuits. Both the amygdala and OFC contain neurons that belong to each network: positive and negative value-coding neurons are present in both areas, and appear to encode the value of cues that signal imminent appetitive and aversive reinforcers, responding in a graded fashion to the value of CSs as well as USs. The dynamics of learning exhibited by appetitive and aversive networks in amygdala and OFC are surprisingly complex, with aversive systems updating faster during reversal learning in the amygdala than OFC, but vice-versa for appetitive networks (Morrison et al., 2011). This suggests that reversal learning is not merely driven by one brain area or the other. The complexity of the dynamics is also illustrated by the fact that the degree to which each area may influence the other is not fixed and instead evolves during the learning process (Morrison et al., 2011) and perhaps in other circumstances as well.

In addition to our neurophysiological findings, behavioral data indicates that the interactions between appetitive and aversive systems are complicated. In a paradigm that required monkeys to make decisions based on the value of stimuli, behavior was sub-optimal when monkeys had to choose between a cue associated with nothing and a cue associated with an airpuff. These results indicate that eye movement choice behavior may be influenced not just by the value of stimuli but also by their salience. It demonstrates that competition between appetitive and aversive networks may occur not only between the values encoded by the two systems but also by the extent to which the systems influence brain structures representing salience, and thereby perhaps generating enhanced attention and eye movements to salient targets.

The complexity of interactions between appetitive and aversive circuits is likely to remain an enduring problem for neuroscientists, but headway is being made. Notably, in our studies of the amygdala and OFC, we have failed to find evidence of anatomical segregation of appetitive and aversive networks (Morrison et al., 2011). Rather, appetitive and aversive networks appear to be anatomically intermingled. Anatomical segregation of these systems would make it easier to develop experimental approaches that can target manipulations of one system or the other to test their causal role in behavior. Fortunately, some recent studies have begun to identify areas where anatomical segregation exists. Two examples of segregation in aversive systems may be found in the work of Hikosaka and colleagues on the habenula (Matsumoto and Hikosaka, 2007, 2008, 2009), and Graybiel’s team in the ACC (Amemori and Graybiel, 2012). The habenula appears to encode negatively valenced stimuli in relation to expectation. The ACC contains networks belonging to appetitive and aversive networks, though there appears to be some anatomical segregation of the aversive network. Both areas are likely to be involved in value-driven decision-making and/or learning. In addition, in contrast to our findings in the monkey, anatomical segregation of appetitive and aversive processing has been observed in the OFC in human fMRI studies (Kim et al., 2006). Our recordings focused only on a restricted part of OFC, largely area 13, and it remains possible that recordings from a more extensive part of the OFC will reveal anatomical segregation of appetitive and aversive systems in the macaque. In general, anatomical segregations may provide more experimentally tractable opportunities for future studies to elucidate details concerning how each network operates.

Despite the anatomical segregation of some aspects of these networks, the challenges ahead are formidable. The amygdala and OFC are two structures intimately related to emotional processing, and these structures, among others, likely mediate the executive control of emotion. Moreover, the amygdala, through its extensive connections to sensory cortex, to the basal forebrain and to the prefrontal cortex is poised to influence cognitive processing. The neurophysiological data we have presented illustrates the complexity of interactions between appetitive and aversive networks. Further, the behavioral data presented suggests that conflict between appetitive and aversive networks extends beyond conflicts about value to conflicts between value and salience. Future studies must clarify how these conflicts are resolved in the brain.

## Conflict of Interest Statement

The authors declare that the research was conducted in the absence of any commercial or financial relationships that could be construed as a potential conflict of interest.

## Authorization for Use of Experimental Animals

All experimental procedures were in accordance with the National Institutes of Health guidelines and were approved by the Institutional Animal Care and Use Committees at New York State Psychiatric Institute and Columbia University.
